# Scalable Production of HPV16 L1 Protein and VLPs from Tobacco Leaves

**DOI:** 10.1371/journal.pone.0160995

**Published:** 2016-08-12

**Authors:** Maryam Zahin, Joongho Joh, Sujita Khanal, Adam Husk, Hugh Mason, Heribert Warzecha, Shin-je Ghim, Donald M. Miller, Nobuyuki Matoba, Alfred Bennett Jenson

**Affiliations:** 1 James Graham Brown Cancer Center, University of Louisville, Louisville, Kentucky, United States of America; 2 Department of Medicine, University of Louisville, Louisville, Kentucky, United States of America; 3 Department of Biochemistry and Molecular Genetics, University of Louisville, Louisville, Kentucky, United States of America; 4 Owensboro Cancer Research Program, Owensboro, Kentucky, United States of America; 5 Biodesign Institute and School of Life Sciences, Arizona State University, Tempe, Arizona, United States of America; 6 Plant Biotechnology and Metabolic Engineering, Technische Universita¨t Darmstadt, Schnittspahnstrasse 3–5, 64287, Darmstadt, Germany; 7 Department of Pharmacology and Toxicology, University of Louisville, Louisville, Kentucky, United States of America; Penn State University School of Medicine, UNITED STATES

## Abstract

Cervical cancer is the most common malignancy among women particularly in developing countries, with human papillomavirus (HPV) 16 causing 50% of invasive cervical cancers. A plant-based HPV vaccine is an alternative to the currently available virus-like particle (VLP) vaccines, and would be much less expensive. We optimized methods to express HPV16 L1 protein and purify VLPs from tobacco (*Nicotiana benthamiana*) leaves transfected with the magnICON deconstructed viral vector expression system. L1 proteins were extracted from agro-infiltrated leaves using a series of pH and salt mediated buffers. Expression levels of L1 proteins and VLPs were verified by immunoblot and ELISA, which confirmed the presence of sequential and conformational epitopes, respectively. Among three constructs tested (16L1d22, TPL1d22, and TPL1F), TPL1F, containing a full-length L1 and chloroplast transit peptide, was best. Extraction of HPV16 L1 from leaf tissue was most efficient (> 2.5% of total soluble protein) with a low-salt phosphate buffer. VLPs were purified using both cesium chloride (CsCl) density gradient and size exclusion chromatography. Electron microscopy studies confirmed the presence of assembled forms of HPV16 L1 VLPs. Collectively; our results indicated that chloroplast-targeted transient expression in tobacco plants is promising for the production of a cheap, efficacious HPV16 L1 VLP vaccine. Studies are underway to develop plant VLPs for the production of a cervical cancer vaccine.

## Introduction

Over 95% of cervical cancers are caused by human papillomaviruses (HPVs). Cervical cancer is the second most common HPV-associated cancer in women, with approximately one-half million new cervical cancer cases and almost 250,000 deaths each year worldwide [1]. The greatest burden of HPV-induced cancers occurs in developing countries. Seventy-five percent of cervical cancers are caused by two HPV types, 16 and 18. HPV16 is also responsible for the majority of anal, penile, vaginal, vulvar, and oropharyngeal cancers [2].

The major recombinant HPV vaccines, Gardasil and Cervarix, are composed of major capsid proteins of HPV which self-assemble into highly immunogenic VLPs *in vitro* and *in vivo* with either T = 7 symmetry, identical to native virions, or T = 1 comprised of 12 capsomers [3–5]. Three VLP-based vaccines have been approved by the FDA to prevent HPV infection: Gardasil (4 HPV types), Gardasil 9 (9 HPV types), and Cervarix (2 PV types). All three vaccines are effective in preventing infection of the HPV types represented in the vaccines and consequently premalignant/malignant disease [6]. Although these commercial vaccines are highly immunogenic, well tolerated, and efficacious, the associated high production and distribution costs have been challenging, particularly for those living in developing countries. To date, no affordable HPV vaccine that can meet the clinical need for under-served populations is available. Therefore, the need for inexpensive and efficient expression systems for production of recombinant proteins has focused the attention of researchers and entrepreneurs on plants as the cheapest potential production hosts.

Plants have emerged as an alternative biotechnological means of producing pharmaceutical [7] and therapeutic proteins [8], with VLPs being the most important candidate vaccines produced in plants [9,10]. Plant-produced VLPs offer many advantages in terms of safety, immunogenicity, and antigen stability together with their cost-effectiveness and scalable production benefits [9]. Research into the development of an effective HPV vaccine using plants has been initiated in several laboratories, including ours, and L1 proteins have been successfully expressed [11–13]. However, all of these studies resulted in very low expression levels of the L1 protein (<1%), except for Maclean et al. [14], who reported the accumulation of L1 that was 11% of total soluble protein (TSP) when the chloroplast signal peptide fused with L1 and formed VLPs in the chloroplasts of plant cells. Since then, various vaccine antigens have been targeted and successfully expressed in chloroplasts, including HPV16 VLPs, at levels up to 24% of TSP [13,15]. It is assumed that greater expression of vaccine antigens in chloroplasts is the result of large gene copy numbers in the plastid genome, or higher stability of proteins in this organelle compared with that of proteins in the cytoplasm or other subcellular compartments [16]. Chloroplast-produced VLPs were reported to be immunogenic in mice and exhibited an effective neutralizing response. However, because of the lack of optimized purification protocols for recovering the intact VLPs, vaccine yield has been low and challenging for clinical trials [17,18].

Another challenge is the chromatographic purification of plant-based proteins. It has been demonstrated that different physiological conditions, together with extraction and purification processes, play a crucial role in denaturation of recombinant protein and determination of yield, stability, and biological activity [19]. CsCl and sucrose gradient methods have been used for purifying L1 VLPs from tobacco plants [13,15]. These methods are cost-intensive and unreliable for industrial and clinical settings. Therefore, it is assumed that chromatographic purification would be the best method to purify L1 VLPs from tobacco plants. Recently, Kim et al. [19] conducted an extensive analysis to purify HPV16 L1 protein from yeast cells using a chromatography system. However, no optimized protocol has been reported for the chromatographic purification of HPV16 L1 VLPs from tobacco plants.

In this study, to express and purify HPV16 L1 VLPs from tobacco plants, we re-designed our expression construct and addressed major shortcomings from previous plant research, including the low yield and less than optimized purification procedures of earlier plant-based technologies. Our improved conditions for HPV16 L1 production in tobacco plants (*Nicotiana benthamiana)* use a high-efficiency binary vector system with a chloroplast signal sequence to assemble the HPV L1 proteins in chloroplasts. The yield of purified plant VLPs was high and antigenically similar to VLPs produced in the baculovirus expression system (Cervarix). Using simple purification steps, this is possibly the first report to present high yield of intact HPV16 L1 VLPs from tobacco plants. We anticipate that our isolated VLPs are safe and can be used as an affordable vaccine worldwide.

## Materials and Methods

### Construction of Vectors

We designed a plant codon-optimized coding sequence for HPV 16 L1 (505 amino acids) based on a strain isolated at the University of Rochester Medical Center (Robert Rose), which is nearly identical to Genbank accession ACV53978.1, except for the occurrence of Ser in place of Thr at position 389. Codons were chosen to optimize expression in tobacco, and avoiding spurious mRNA processing signals [20]. The C-terminal truncation to remove 22 amino acids and add a stop codon and *Bam*HI site was obtained by PCR with the end-tailoring primer 16L1d22-Bam-R (5’–GGGGATCCTTATCCTAATGTGAACTTTGGCTT). For chloroplast targeting, a 59 amino acid plastid peptide (TP) coding sequence from *rbcS* was fused to the initiation codon of the L1 gene using overlap extension PCR. The plastid targeting sequence was amplified from pICH12190 (Icon Genetics GmbH, Halle, Germany) using the primers TP-Xho-F (5’-ccctcgagaacaatggcttcttctatgctttcttc) and TPL1-OL-R (5’-GAAGGGAGCCAAAGAGACATGCATTGGACTCTTCCTCCGT), while the 5’ end of the L1 gene was amplified using primers TPL1-OL-F (5’- acggaggaagagtccaatgcatgtctctttggctcccttc) and 16L1-447-R (5’- cacaactgtgtttgcttgtaatcc). The two overlapping fragments were mixed and amplified using the terminal primers TP-Xho-F and 16L1-447-R, and the resulting product was digested with *Xho*I and *Nco*I and ligated with pIBT210.1 [20], digested likewise to produce pTPL1-210. The TPL1 segment was fused to the 3’ end of the L1 gene by ligation at the *Nco*I site. The L1 genes were end-tailored by PCR with *Bsa*I sites to allow insertion into pICH26212 (Icon Genetics), yielding p26212-16L1d22 (483 aa L1, cytosol-targeted), p26212-TPL1d22 (483 aa L1, chloroplast targeted), and p26212-TPL1F (505 aa L1, chloroplast targeted).

The *E*. *coli* and *Agrobacterium* transformed plasmid DNA was confirmed for integration using *Bam*HI and *Xho*I restriction digestion analysis and the PCR amplification method. The set of primers used were MZ-1L (5'-GCAGGTGTGGTTGACGAAT-3') and MZ-1R (5'-CAAACGTGCGTTAACAGGTG-3') (Integrated DNA technologies, USA) for amplification of N- and C- termini of the target gene, respectively.

### Electroporation and Agrobacterial transformation

Initially, *A*. *tumefaciens* (GV3101) cells were made electro-competent [21], and 50 μL of cells were mixed with 50–200 ng of the purified respective HPV16 L1 DNA in a 1.0 mm gap electroporation cuvette (Molecular Bio Products, USA). The cells were transformed using a GenePulser (BioRad, USA) with a setting of 2000V for 5 ms. Electroporated cells (51 μL) were immediately added to 1 mL YenB broth (yeast extract and nutrient broth, pH 7.0) containing Kanamycin (50 μg mL^-1^) and incubated with shaking for 1 h and then plated onto YenB media containing appropriate antibiotics.

### Infiltration of tobacco leaves

Agro-infiltration of tobacco leaves was completed with transformed *Agrobacterium* cultures containing integrated HPV16 L1 genes. Cultures were grown from starter cultures with shaking at 28°C to attain the exponential phase (optical density at 600 nm [OD_600_]; 0.6 to 0.8) in YenB broth containing the appropriate antibiotics. The bacteria were pelleted down by centrifugation at 3000×*g* for 10 min, suspended in infiltration buffer (10 mM MES and 10 mM MgSO4, pH 5.5). Final OD_600_ after centrifugation and resuspension was 0.1. The vacuum infiltration technique was employed to infiltrate 2–4 week old leaves of *N*. *benthamiana* plants. Following infiltration, the plants were grown for 1–9 days post infiltration (DPI) under conditions of 16 h light, 8 h dark at 23°C.

### Preparation of leaf extract

In the preliminary analysis, 200 mg of tobacco leaves were harvested at 6 DPI and proteins were extracted using HEPES buffer as described [13]. For time point analysis, 200 mg of fresh tobacco plant leaves were harvested at 3–9 DPIs and homogenized at the ratio of 1:2 in relation to the volume of respective buffers (400 μL; pH 2–8 with or without 0.1 M NaCl and 40 mM ascorbic acid) in a Stormblender (Bullet blender Storm 24, Next Advance, USA) to extract the best proteins. The lysates were clarified at (14,000×*g*) for 10 min at 4°C. The supernatant collected after clarification was used for further analysis.

### SDS-PAGE and Immunoblot analysis

The extracted soluble protein samples (supernatant) collected from homogenized and clarified tobacco leaves were mixed with sodium dodecyl sulfate (SDS) loading buffer (20% glycerol, 4% SDS, 100 mM Tris, pH 6.8, 0.002% bromophenol blue), and heated at 95°C for 5 min. These proteins were separated on SDS-PAGE (sodium dodecyl sulfate-polyacrylamide gel electrophoresis) gels (NUPAGE 4–12% Bis-TRIS, Invitrogen, USA). Gels were either stained with coomassie blue (SimplyBlue SafeStain; Invitrogen, USA) or transferred to a PVDF membrane (Immobilon-P transfer membrane, EMD Millipore, USA) and saturated with 5% milk in Tris-buffered saline and Tween (TBST) for 1 h. The membrane was then incubated overnight at 4°C with HPV16 L1 specific monoclonal (16E, in-house) or polyclonal (disCPV2, in-house) antibodies with 3% milk/TBST (Tris and NaCl containing 0.1% Tween 20). The membrane was washed three times with TBST and finally incubated with 1:5000 diluted secondary antibody (peroxidase conjugated goat anti-mouse IgG, Thermo Scientific Pierce, USA). Proteins were detected by chemiluminescence (SuperSignal West Dura Extended Duration Substrate; Thermo Scientific Pierce, USA) and bands were visualized on x-ray film (CL-XPosure Film, Thermo Scientific Pierce, USA) using an SRX-101A processor (Konica Minolta Medical Imaging USA, Inc). Baculovirus-infected insect cell VLPs made in-house was used as a positive control. The purified fractions from CsCl, ammonium sulfate (AMS), size exclusion chromatography (SEC), and ion-exchange chromatography (IEC) fractions were also analyzed for protein expression, as described above.

### Antibodies

16A and C are murine monoclonal antibodies made in the laboratory against intact virus-like particles and characterized [22] whereas 16E was generated to detect sequential epitopes of L1, using wild type HPV16 (antigen produced in insect cells), respectively. DisCPV2 is a rabbit polyclonal antibody made against disrupted canine papillomavirus produced using xenograph system. 1H8 is the broad spectrum mouse monoclonal HPV antibody which was produced against SDS-disrupted bovine papillomavirus type 1 (BPV1) and used to identify the product of the L1 open reading frame (ORF) of BPV1. IH8 was found to be reactive with purified major capsid protein (L1) and known to detect the FGA epitope of both BPV1 and HPV16, respectively.

### ELISA

The ELISA was performed on all homogenized, purified leaf extracts and sera collected from immunizing mice as described in sections 2.4, 2.5 and 2.14. The 96-well ELISA plates (Immunolon 2; Dynatech) were coated overnight at 4°C with 100 μL of plant extracts or 500 ng per well of purified plant VLPs re-suspended in 1X Dulbecco's phosphate-buffered saline (DPBS). 500 ng of baculovirus-infected insect cell VLPs were loaded as a positive control. The plates were washed three times with washing buffer (PBS-T) and blocked with 5% BSA in 1XPBS (PBSA) for 1 h at 37°C. After washing three times, plant extracts coated plates were incubated with monoclonal or polyclonal antibodies against HPV16 L1 conformational or sequential epitopes, at a dilution of 1:1000 in 1% PBSA for 1 h at 37°C. Similarly, the purified plant VLPs coated plates were reacted with collected mouse sera, at a dilution of 1:100 in 1% PBSA for 1 h at 37°C. Moreover, to test the immunogenicity of plant derived VLPs, pooled mice sera were diluted 4-fold in 1% PBSA (1:50–1:51200) and titrated against 500 ng of insect cell derived VLPs. Thereafter, plates were washed thrice with PBS-T, and incubated with respective alkaline phosphatase-conjugated goat anti-mouse or anti-rabbit IgG (H + L) (Sigma, USA) at a dilution of 1:5000 in 1% PBSA for 1 h at 37°C. Unbound secondary antibodies were removed by washing and bound antibodies were stained with an alkaline phosphatase chromogenic substrate (Sigma, USA), and absorption was measured at 405 nm. One percent PBSA and sera collected from unimmunized mice were used as negative controls for the background reaction.

### Protein purification by cesium chloride density gradient ultracentrifugation

Freshly harvested tobacco leaves (5 g) were grounded in liquid N_2_ and homogenized with extraction buffer (PBS with 40 mM ascorbic acid, pH 7.0). The lysate was centrifuged at (14,000×*g*) for 30 min at 4°C. The cleared supernatant was collected and mixed with cesium chloride (CsCl) solution to a concentration of 1.31 g mL^-1^ as determined using a refractometer (Leica Mark II, Reichert-Jung) and centrifuged at (122,800×*g*) for 20 h. The bands on CsCl gradients were collected, their densities measured, and the CsCl solution was replaced with 1XDPBS containing CaCl_2_ and MgCl_2_ (Life Technologies) by dialysis. The dialyzed sample was again centrifuged for 2 h at the same speed to pellet down the VLPs, which were then suspended in a minimal amount of 1XDPBS for storage at -80°C until analysis.

### Ammonium sulfate precipitation and removal of contaminated proteins

To remove contaminants from L1 proteins, ammonium sulfate precipitation was conducted as previously described [19]. The precipitated protein was pelleted down at (23,700×*g*) for 10 min at 4°C. The pelleted protein was re-suspended with PBS+ 0.01% Tween 20, and the re-suspended solution was stored at 4°C.

To remove further contaminants, the ammonium sulfate precipitate was dialyzed against PBS + 0.01% Tween 20 [19]. Using the previously described method [19], the precipitated protein was removed by centrifugation at (23,700×*g*) for 10 min. The obtained supernatant was used for the purification process.

### Ion exchange chromatography

The supernatant obtained after ammonium sulfate precipitation was dialyzed against the binding buffer (PBS containing total 0.1 M NaCl, pH 7.2 + 0.01% Tween 20) for 3 h at 4°C. The 5 mL cation exchange column (Hitrap sepharose [CMFF], GE Healthcare Life Sciences, USA) was used to purify the L1 protein. The column was equilibrated with binding buffer, and the dialyzed sample (5 mL) was loaded onto the column. The column was then washed with five column volumes of binding buffer with a flow rate of 0.4 mL min^-1^, followed by the elution buffer PBS containing 2.0 M NaCl (salt gradient) and 0.01% Tween 20. The total run lasted 1.5 h. The fractions corresponding to peaks were pooled and dialyzed against binding buffer and concentrated with an additional 2-h centrifugation step (122,800×*g*).

### Size exclusion chromatography

SEC was performed on dialyzed plant VLPs purified by the CsCl density gradient ultracentrifugation method and ammonium sulfate precipitation. Dialyzed samples were loaded on (16/600) Superdex column (Hitrap, GE Healthcare Life Sciences, USA). The running buffer for this column was PBS, pH 7.2, 0.01% Tween 20. Fractions were collected from the column at 0.5 mL min^-1^ for 4 h; their absorbance at 280 nm was measured with a spectrophotometer, and HPV16 L1 protein was analyzed by SDS–PAGE and immunoblotting, as described above.

### Determination of protein concentration

Total protein concentration of each VLP preparation and ammonium sulfate precipitated samples was determined using a Nanodrop ND-8000 (Thermo Scientific, USA) at 280 nm and the Bio-Rad Bradford protein assay reagent (Bio-Rad Laboratories, USA) with bovine serum albumin (BSA; Pierce, USA) as a standard. The yield of L1 protein was quantified using SDS-PAGE analysis. Serially diluted HPV16 L1 VLPs from leaves and insect cells were loaded on an SDS-PAGE gel, and their band densities were measured by Quantity one image analyzer (version 4.5, Bio-Rad).

### Electron microscopy

The dialyzed, concentrated and purified HPV16 L1 VLPs were absorbed onto carbon-coated grids (Electron Microscopy Sciences, USA) and negatively stained with 2% phosphotungstic acid (pH 6.8). The quality of VLPs was examined at a final magnification of 41,000× by transmission electron microscopy (Phillips CM-12).

### Mice

C57BL/6J mice were obtained from the Jackson Laboratory (Bar Harbor, ME).

Only female mice were used to minimize fighting. Mice were maintained in humidity-, temperature-, and light cycle (12:12) controlled vivarium under specific pathogen-free conditions. Mice were housed in double-pen polycarbonate cages (330 cm^2^ floor area) at a maximum capacity of four mice per pen. Mice were allowed free access to autoclaved food (NIH 31, 6% fat; Lab Diet 5K52, Purina Mills, St. Louis, MO) and acidified water (pH 2.8–3.2). Mice were euthanized using CO_2_ in a chamber fitted with an appropriate pressure-reducing regulator and flow meter or equivalent equipment to ensure a gradual displacement of 10–30% volume/minute. All studies were done with the University of Louisville, Institutional Animal Care and Use Committee (approval number #13051) and Biosafety Committee (approval number IBC #10–024) approvals.

### Mouse immunization

In order to collect hyperimmune sera of plant VLPs, C57BL/6J wild-type mice were immunized as previously described [23]. Hundred μg of purified plant VLPs were prepared for each injection without any adjuvant application and administered intraperitoneally to C57BL/6J mice for three times at 2 weeks of interval. Blood was drawn from unimmunized (control) and immunized mice and sera were separated by centrifugation at 3,000 rpm for 10 min and stored at 4°C.

### Statistical analysis

All statistical analyses were performed with GraphPad Prism statistical software (version 4.03; La Jolla, CA). Statistical differences were determined by Student's t-test and one was ANOVA. Differences were considered to be statistically significant if the p-value was 0.05.

## Results

### Infiltration of tobacco plants with HPV16 L1 DNA

**Fig 1:**
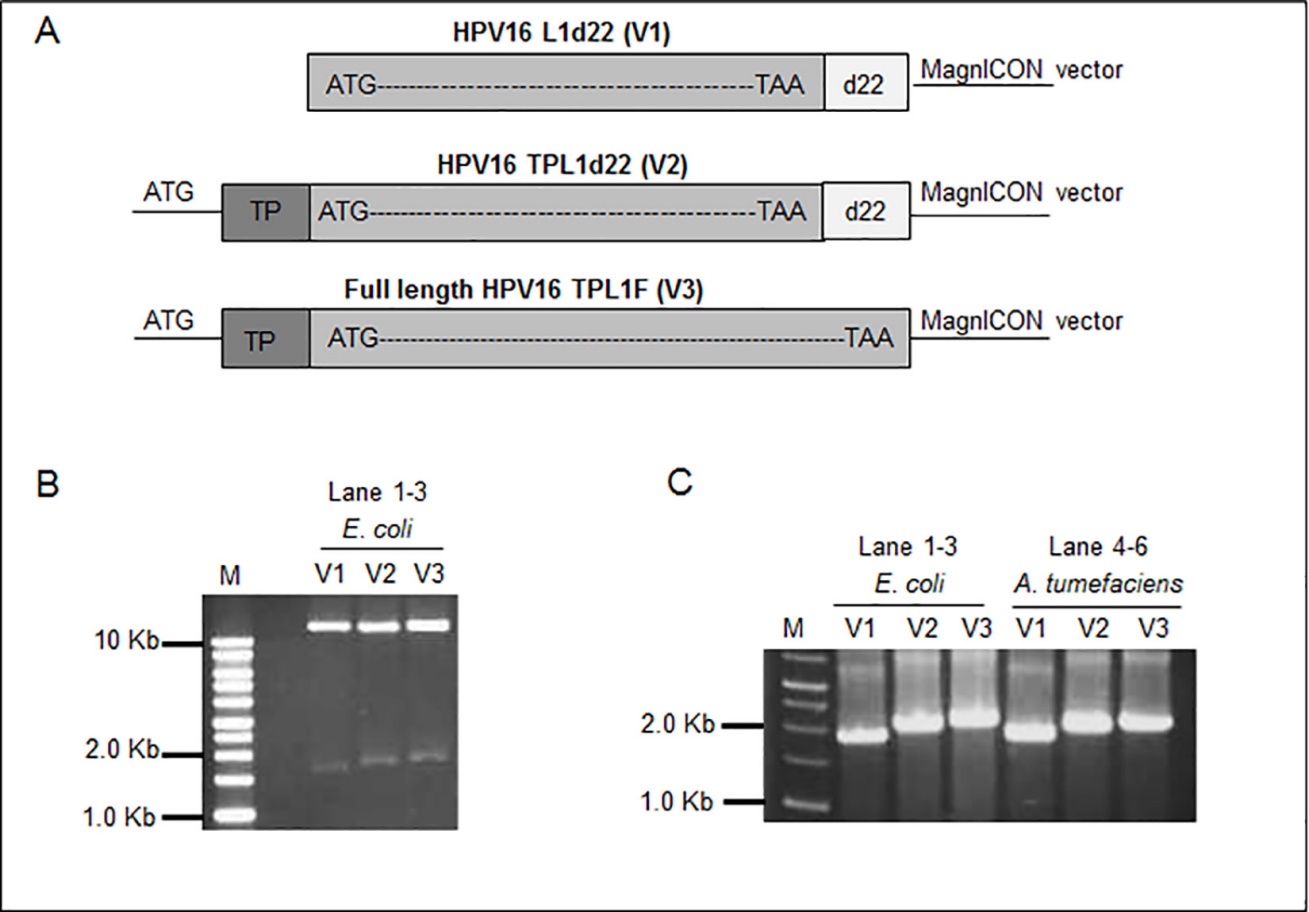
Diagrammatic presentation of constructed protein expression vectors for transformation of *Agrobacterium* and plants. **(A)** Pictorial representation of constructed vectors. Restriction digestion profile of cloned plasmids DNAs (V1, V2, and V3) from *E*. *coli* (Lane 1–3) **(B)** and PCR amplifications of transformed *E*. *coli* (Lane 1–3) and *Agrobacterial* cells (Lanes 4–6), **(C),** respectively Target DNAs (1.5 and 1.7 Kbs) were found on 1% agarose gel when compared with DNA molecular weight marker (Lane M, 1 kb DNA ladder, Invitrogen).

Three constructed vectors p26212-16L1d22, p26212-TPL1d22, and p26212-TPL1F for protein expression in plants were designated as V1, V2, and V3, respectively (Fig 1A). Restriction digestion and PCR amplification profiles were determined in Fig 1B and 1C, respectively.

### Expression of HPV16 L1 protein

**Fig 2:**
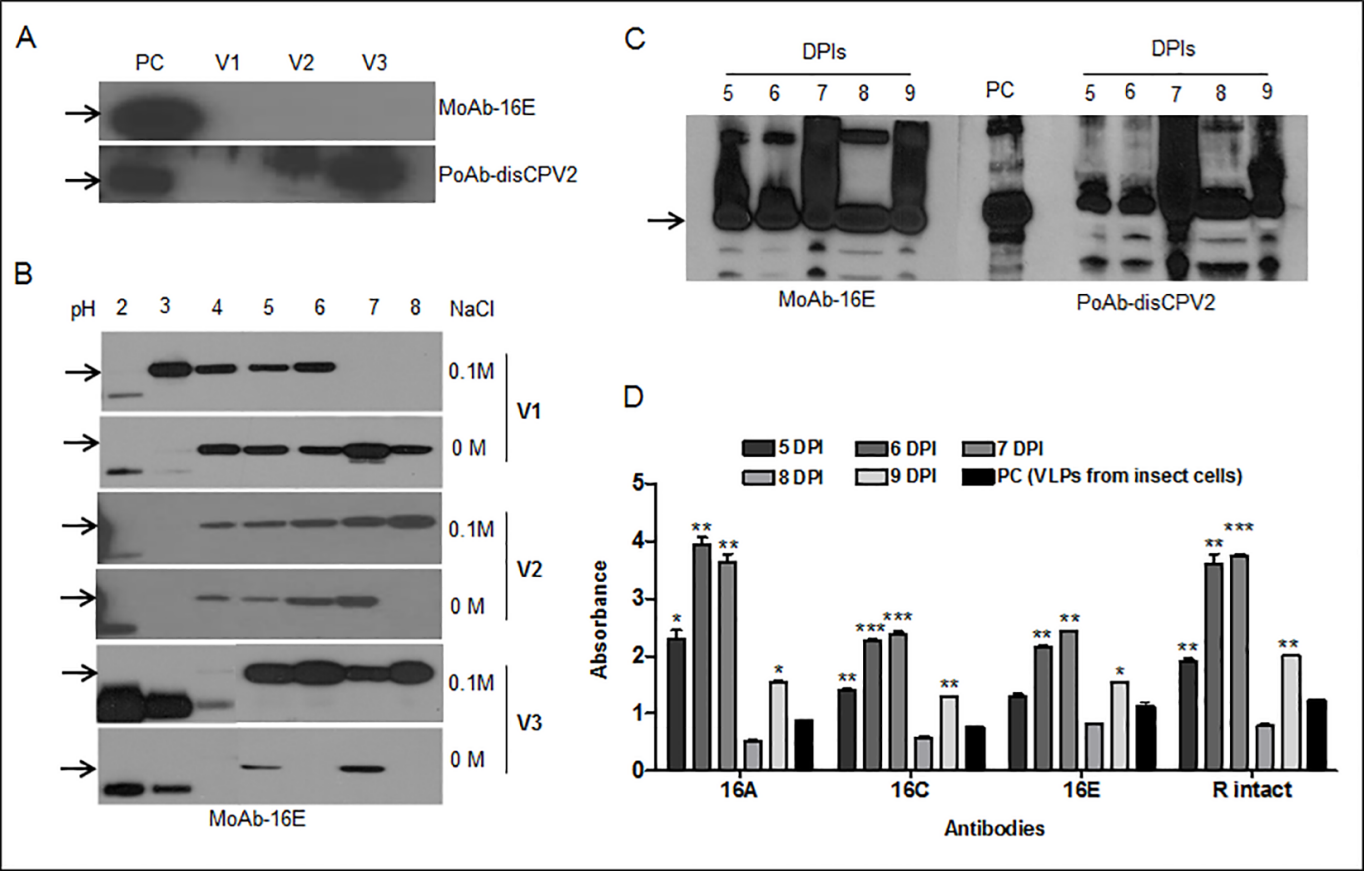
The detection profile and quantification analysis of L1 protein extracted from transformed tobacco plants. The immunoblots represent the HPV16 L1 detection profile of different vector-infiltrated plants extracted with **(A)** HEPES buffer, **(B)** using different physiological conditions with pH 2–8 in the presence (WS) or absence of salt (NS), **(C)** extraction of HPV16 L1 protein with PBS buffer at 5–9 days post infiltration (DPI) from plant leaves infiltrated with construct (TPL1F; V3). Antibodies, MoAb-16E and PoAb- disCPV2 were used for immunoblots. The positive control (PC) was disrupted VLPs from insect cells and arrows in immunoblots show HPV L1 band at 56 kDa. **(D)** Bar diagram of ELISA for VLPs extracted from crude plant extract (V3) at 5–9 DPIs. MoAbs-16 A, C and E and PoAb-R intact were used to detect specific sequential and conformational epitopes of HPV16 L1 and VLPs from insect cells were used as a positive control (PC). Student’s t-test was done to compare VLPs from plant extract at 5–9 DPIs with VLPs from insect cells. *p < 0.05; **p <0.01 and ***p <0.001.

Three steps were followed to optimize the expression level of the L1 protein. At first, the expression level of the L1 protein in plants by different expression vectors was confirmed at 6 DPI. Extracted L1 proteins (L1d22, TP+L1d22, and TP+L1F) were detected at target size of 51 or 56 kDa when compared with baculovirus-infected insect cells used as a positive control (Fig 2A). Although no target L1 protein was detected using monoclonal antibody (16E), the L1 protein from p26212-TPL1F (V3) vector was extracted at the highest level among the three constructs using polyclonal antibody (disCPV2).

Secondly, to optimize and characterize the purification condition for the L1 protein extracted from infiltrated plant leaves, different physiological buffers with various pH (2–8), ascorbic acid supplement, and salt (0 and 0.1 M) were used. Proteins were successfully detected in crude extracts homogenized with pH 4 to 8 buffers, and higher concentrations were found in the presence (0.1 M) rather than the absence of salt containing buffers (Fig 2B). Overall, the neutral phosphate buffer (pH 7) with (0.1 M) salt was chosen as the best extractable solvent. Among these three constructs, V3 was highly extracted and selected for further experiments.

Thirdly, a time point study was conducted to determine the optimal duration of protein expression in V3 transformed tobacco plants. Interestingly, the expression levels of the L1s were high at 5 to 9 DPIs, whereas non-infiltrated control leaves were negative for all the tested antibodies (S1 Fig). The protein was expressed maximally at 7 DPI, and it decreased as depicted in Fig 2C. Moreover, the highest expression of epitopes was found in sample extracts between 6 and 7 DPI plants (Fig 2D).

Based on obtained results, 6 DPI leaves transformed with V3 vector and extracted with neutral PBS buffer was chosen for further purification and quantification studies. Compared with purified VLPs from insect cells, >2.5% of TSP for L1 proteins (2.5 μg L1 / 100 μg TSP) was calculated and overall, 250 mg of L1 yield was obtained per kg of plant biomass.

### Purification of HPV16 VLPs

**Fig 3:**
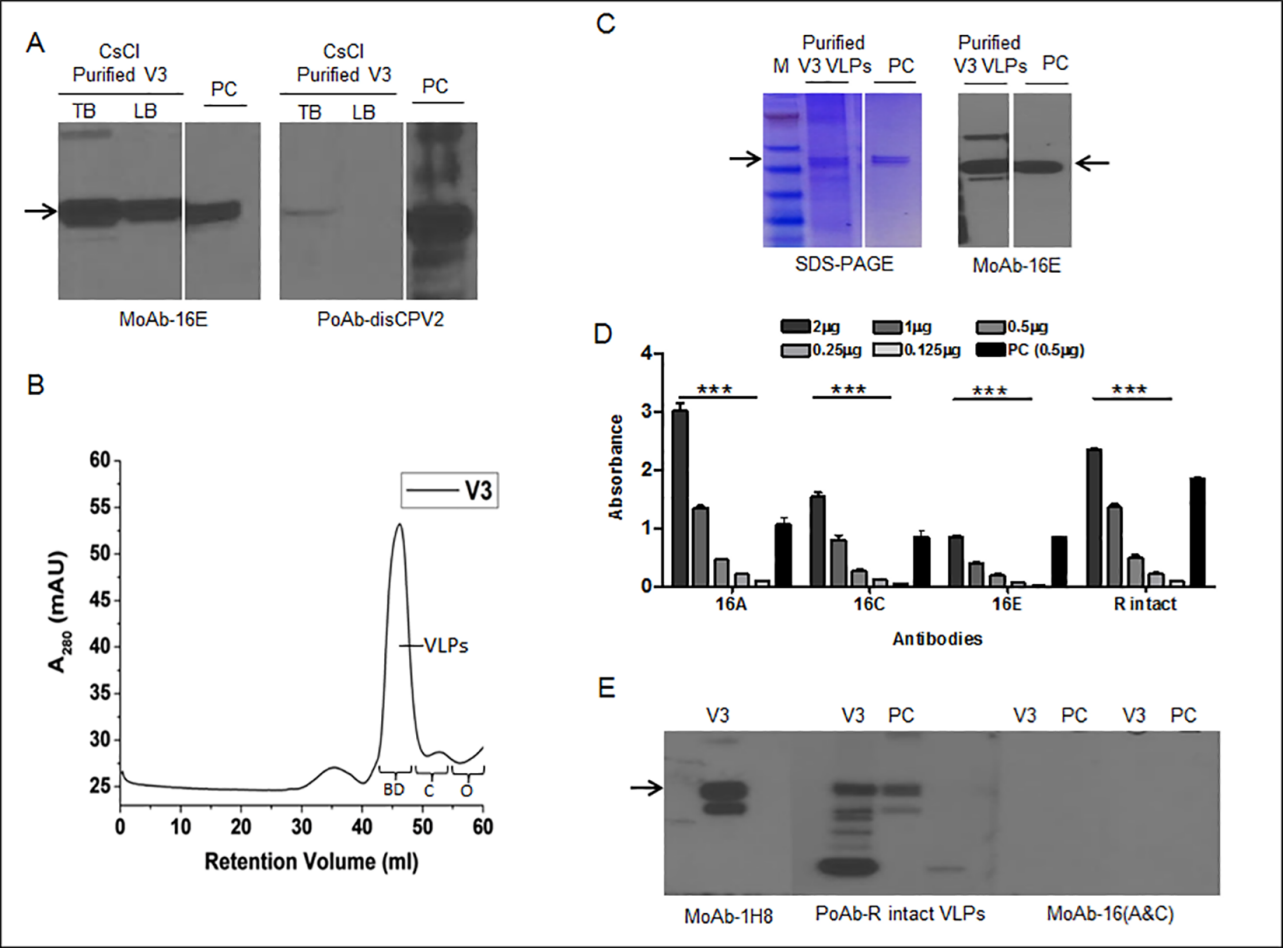
Purification profile of V3 infiltrated tobacco plants. **(A)** Immunoblot showing the target L1 protein collected from CsCl density gradients top (TB) and lower bands (LB) from V3 infiltrated plants. **(B)** The size exclusion chromatogram of CsCl collected and dialyzed V3 sample on 16/600 superdex-200 prep grade column using FPLC system. The molecular standards represented on the chromatogram are BD; bluedex (2000 kDa), C; conalbumin (75 kDa), O; ovalbumin (43 kDa). **(C)** SDS-PAGE and immunoblot showing the L1 band of dissociated chromatographic purified VLPs. The VLPs from insect cells were used as loading positive control and arrows in immunoblots show HPV L1 band at 56 kDa; 16E was used as HPV16 L1 sequential epitopes detecting monoclonal antibody. **(D)** ELISA profile for characterization of plant purified HPV16 VLPs using monoclonal (16A, C, and E) and polyclonal (R intact) in-house antibodies. 16A and C detect conformational epitopes, whereas 16E and R intact detect both conformational, as well as sequential epitopes. (**E)** Validation of purified VLPs by immunoblot using the same sequential and conformational antibodies as described in D. One way analysis of variance (ANOVA) was done to appreciate the dose response and a value *p < 0.05 was considered significantly different.

When crude extracts were purified using CsCl gradient centrifugation, two bands were visualized at approximately 1.29 g/cm^3^ density in centrifuge tubes and were referred to as the top and lower bands. L1 proteins were detected in both the collected bands, however, a relatively stronger signal was identified from the top collected band than the lower one (Fig 3A). In addition, the presence of conformational epitopes on the VLP's matrix was analyzed by ELISA assay and compared between the two collected band samples (data not shown). The results showed that top collected band displayed higher ELISA signals than the lower band, suggesting more accumulation of VLPs in the top band.

In order to remove residual contaminants and clarify the high molecular size of VLPs, the dialyzed top band of L1 was further subjected to size exclusion (SEC) column. Differently assembled forms of L1 VLPs were eluted in a peak with an elution volume of approximately 42 mL of running buffer between 2000 and 75 kDa when compared with different molecular weight protein standards (Fig 3B). L1 proteins with approximately 56 kDa size (Fig 3C) were detected from the pooled fractions (#44–54) of SEC. collected fractions were analyzed by an ELISA plate and showed a high signal for conformational epitopes (particularly at 2.0 and 1.0 μg of loaded plant VLPs) when compared with a positive loading control, insect cell VLPs (0.5 μg) (Fig 3D). When ELISA results were further confirmed by immunoblot assay, polyclonal antibody (R intact) and a monoclonal antibody recognizing FGA epitope (1H8) detected an L1 band at 56 kDa, as shown in Fig 3E. However, no L1 protein was detected in either plant or positive control using HPV16 conformational antibodies (16A and C) which exhibited a high signal in the ELISA assay, likely because the critical epitope conformation had been lost under the denaturing conditions (Fig 3E).

In order to optimize the conditions and strategy for collecting the accumulated VLPs, alternative purification methods were tested. Using forty-five percent of AMS, most of the L1 proteins and VLPs were successfully recovered. Following each step of the AMS treatment, TSPs were precipitated and clarified, which drastically reduced the TSPs to one-fifth of 760 mg (152 mg).

**Fig 4:**
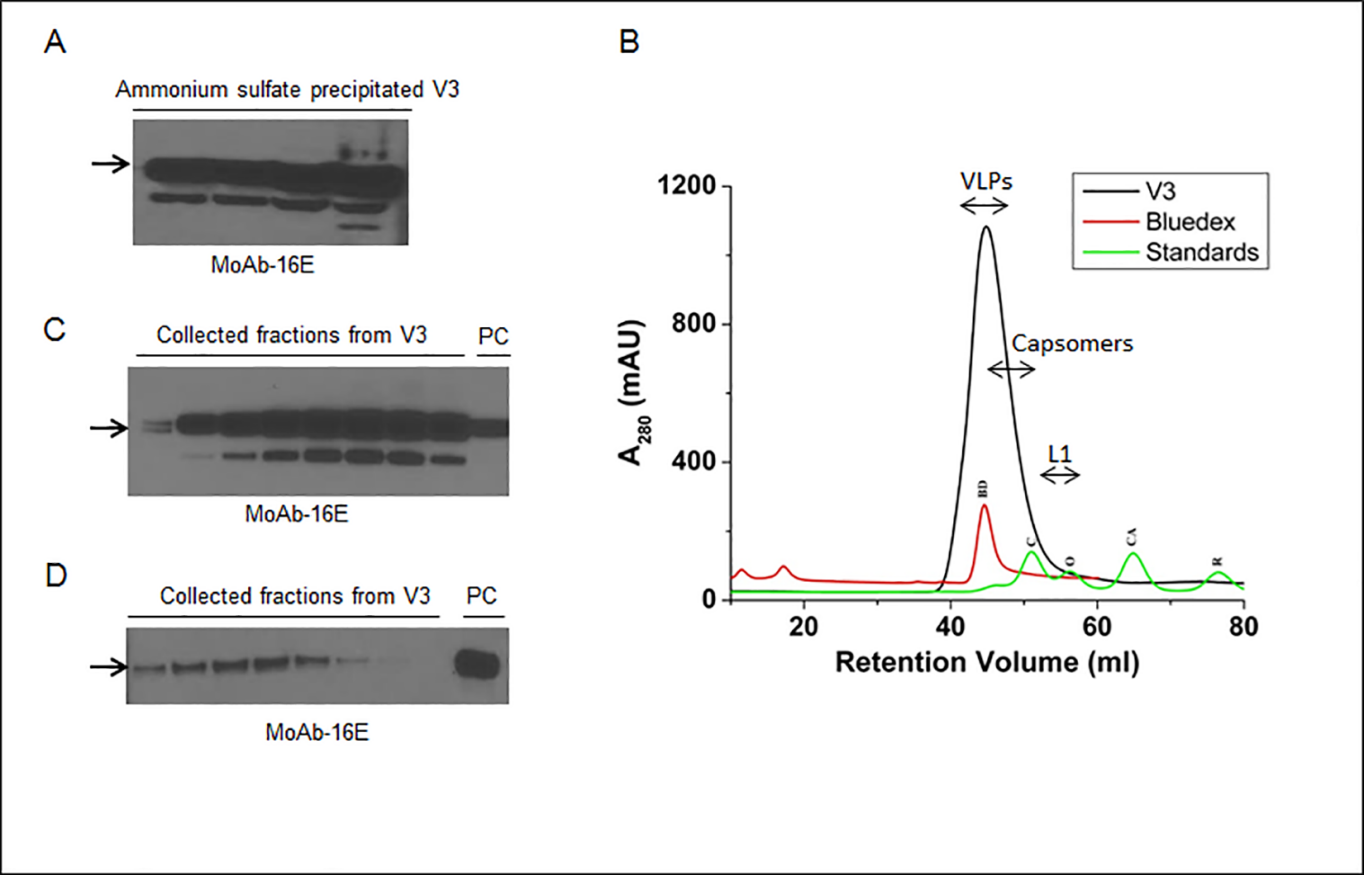
Ammonium sulfate precipitation and purification of the V3 infiltrated plants. **(A)** Immunoblot of ammonium sulfate precipitated L1 protein samples collected at various stages of purification. 16E monoclonal antibody was used to detect the target L1 protein. **(B)** The size exclusion chromatogram for purification of ammonium sulfate precipitated L1 protein and VLPs using FPLC system. The running profile of molecular standards, BD; bluedex (2000 kDa), C; conalbumin (75 kDa), O; ovalbumin (43 kDa), CA; carbonic anhydrase (29 kDa) and R; ribonuclease (6.5 kDa) are showed on the chromatogram. **(C)** Immunoblot showing the detection profile of fig B and L1 protein was detected in the collected fractions corresponding to 40–50 mL fraction size. **(D)** Immunoblot showing the bands of cation exchange chromatography collected L1 protein. Sample (V3) and purified VLPs from insect cells (PC) were eluted at 0.3 M and ≥ 0.7 M NaCl, respectively. The arrows in immunoblots show HPV L1 band at 56 kDa.

These results indicated that more than 75% of TSPs were effectively precipitated; most of the recovered L1 proteins were soluble in PBS buffer, as shown in Fig 4A. When an additional step of purification was conducted using SEC, the AMS precipitated L1 proteins showed an intense (1000 mAU) single symmetric peak with 38–55 mL of elution buffer (Fig 4B), and recovered fractions (# 40–50) showed the presence of the L1 protein revealed by immunoblot assay (Fig 4C).

Based on the isoelectric point (pI) of 8.5 for the L1 protein, crude extracts, CsCl, and AMS precipitated protein samples were also subjected to CM Sepharose Fast Flow, a weak cation exchanger. We found that plant protein samples showed low binding affinity with the cation column and started eluting early with only 0.3 M of NaCl. Unlike the plant protein, the insect cell-expressed L1 protein was eluted at ≥ 0.7 M NaCl (data not shown). These results were also verified by immunoblot assay, which showed that most of the plant L1 protein was eluted without being bound and lost during the wash (Fig 4D).

### Electron microscopic and immunogenicity analysis of plant VLPs

**Fig 5:**
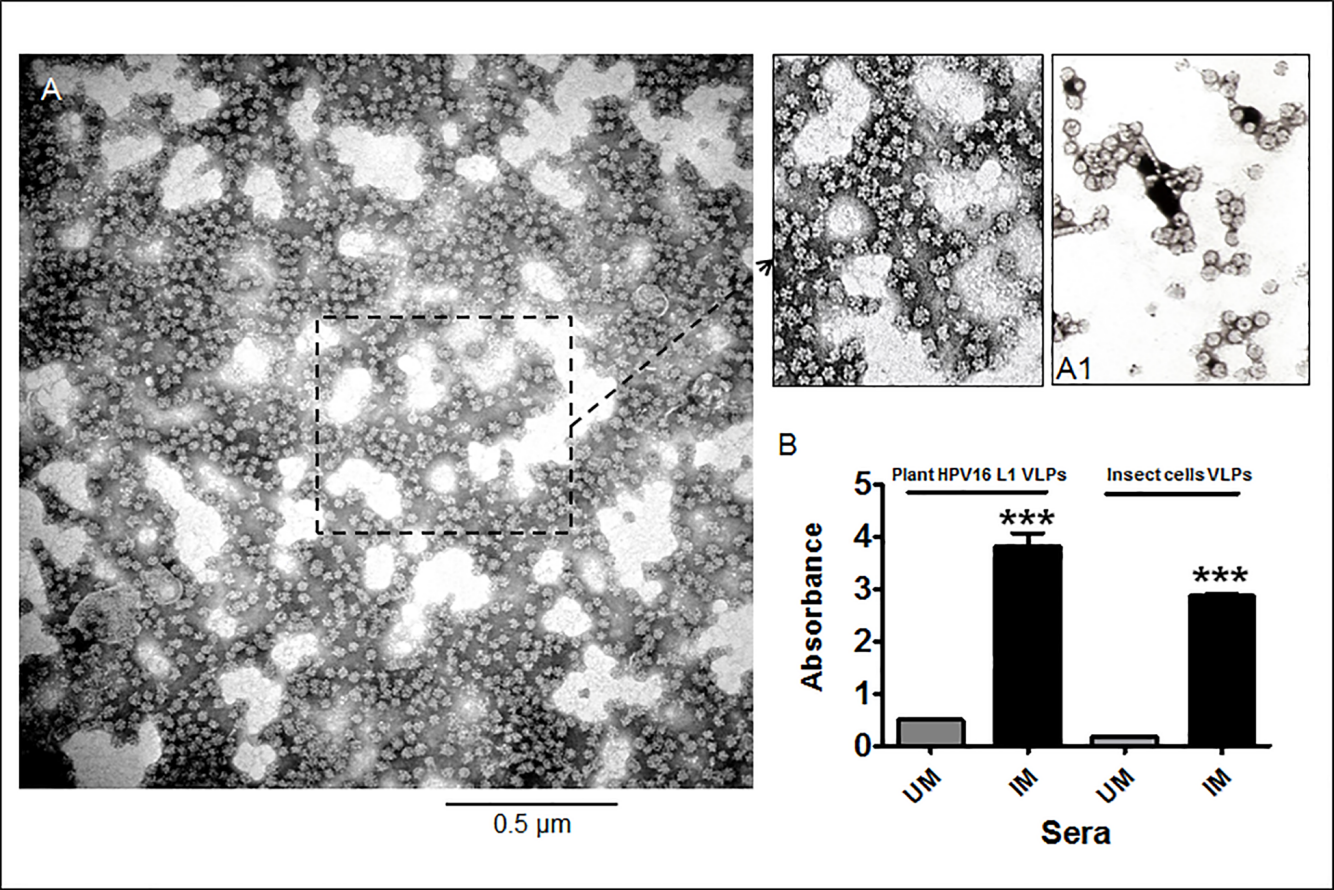
Electron micrograph and immunogenicity profile of purified plant HPV16 L1 VLPs. Purified (V3) sample **(A)** and HPV16 VLPs derived from insect cells (**A1**) were absorbed on carbon coated grid and negatively stained with 2% phospho-tungstic acid (pH 6.8). Magnification was 46,000x; **(B)** The ELISA profile of hyperimmune sera collected from plant HPV16 L1 VLPs immunized mice. First two bars show the generated immune response against the sera of unimmunized mice (UM) act as a control and immunized mice with plant purified HPV16 VLPs, respectively when plant VLPs were loaded as antigens onto ELISA plate. Third and fourth bar represents the insect cell VLPs response, coated as antigens, against the sera collected from the above unimmunized (UM) and immunized mice (IM) with plant VLPs, respectively. Student’s t-test was done to compare differences between unimmunized (UM) and immunized mice (IM) from VLPS of plant extract and insect cells.

Our results confirmed that purified HPV16 L1 protein self-assembled into stable VLPs in plants. As shown in Fig 5A, fractions recovered after SEC and CsCl purification methods were the best and showed numerous VLPs, predominantly 35–55 nm in size. The top and bottom bands from the CsCl density gradient contained a lower density of partially purified VLPs. However, AMS purification followed by SEC gave the highest yield of L1, but an electron microscopic analysis showed only the presence of L1 capsomers (data not shown). In contrast, cation exchange collected L1 did not show the formation of VLPs because most of the L1 protein was lost during the purification process.

To test the immunogenicity of plant VLPs, antibody titers were evaluated in immunized mice (Fig 5B), where 1:100 diluted sera of mice immunized with the plant VLPs showed high reactivity with both plant and insect cell VLPs coated plates while no anti-VLP antibody response was detected in unimmunized mice of both the sets. Also, when plant VLPs immunized sera was titrated against insect cell VLPs, high antibody titers (>3200) was observed.

## Discussion

The aim of our study is to successfully express and purify HPV16 L1 VLPs from tobacco plants using MagnICON expression system. The L1 protein was designed to be accumulated in chloroplasts, which lead to the assembly of predominantly 35–55 nm VLPs with maximal protein level (>2.5% of TSP).

Low levels of recombinant HPV L1 protein expression lead to decreased formation of VLPs, which in turn reflects the total outcome yield of purified VLPs [19,24]. Therefore, the nature of L1 expression and the extent of recovery of the target protein during downstream processing are important factors that affect costs in the production of biopharmaceuticals based on recombinant proteins [25]. With the substantial strides of HPV16 VLPs as a promising vaccine platform [26], the major challenge is to develop production platforms that can deliver VLP-based vaccine to clinics timely with a reduced price [9]. Although, the development of plant virus-based transient plant expression systems has overcome the challenges of L1 expression and yield [27,28], studies to encapsulate HPV VLPs are still in progress.

Compared to nuclear transformation, it has been documented that genetic engineering of plastids offers many advantages, which make the system ideal for applications involving plant-made pharmaceuticals [29]. These include high levels of transgene expression because of a large number of copies, absence of epigenetic effects and transgene containment via maternal inheritance, and multi-gene expression in a single transformation event [30–32]. Subsequently, a variety of adjustments was made to increase the yield and expression of proteins in chloroplasts. Many vaccine antigens against several human and animal diseases were successfully expressed in chloroplasts [32]. Recently, HPV16 L1 proteins were successfully expressed in chloroplasts, although no significant data related to their optimal yield was obtained [13,33]. Accordingly, we used chloroplast transit peptide (TP) in this study to induce L1 expression with a magnICON vector (p26212). The TP sequence was added to the L1 primary sequence after eliminating the original nuclear-translocated localization signal (NLS). Therefore, we have a truncated L1 without 22 amino acids of the C-terminal end that corresponds to the localization signal. In order to analyze the effect of transition signals, three different vectors were constructed (p26212-16L1d22, p26212-TPL1d22 and p26212-TPL1F) and compared. Our approach based on the use of a chloroplast transit peptide and a magnICON expression vector successfully led to a higher expression and accumulation of VLPs in the chloroplasts of plant cells.

Expression of L1 proteins was shown in plants transfected with all three constructs upon extraction with an HEPES buffer [13], and the highest level was exhibited in plants transfected with the p26212-TPL1F vector. The lowest expression was shown where no transit signal peptide (p26212-16L1d22) was used. Although the cellular mechanism is not clearly understood for the protein expression process, the existence of any localization signal induced higher yield, and the existence of both TP and NLS seem to increase the yield. The constructed vector with full-length L1+TP was the best for L1 protein expression, and therefore, we decided to use the p26212-TPL1F vector for L1 expression in plants. However, L1 protein levels in HEPES buffer extracts of all three vectors were not high enough to continue for VLP purification.

Low yield of L1 in extracts with HEPES buffer suggested that L1 protein might be degraded during the extraction and homogenization process. To overcome the problem of low yield, we optimized our extraction protocol using different buffers and salts, and an antioxidant supplement was added to reduce L1 oxidation. Our results indicated that extraction of HPV16 L1 from leaf tissue was most efficient (>2.5% of TSP) with a low-salt phosphate buffer (pH 7.0). Using this neutral pH phosphate buffer, we found that L1 expression reached the maximum between 6 and 7 DPI. A study had previously shown that neutral or lower pH aided in the assembly of T = 1 or T = 7 L1 VLPs [3]. Similarly, our expression level analysis suggested that 6 DPI, with low salt and neutral pH, was the best condition for harvesting and extracting proteins.

Notably, our improved purification protocol gave higher L1 VLP's recovery with the successful removal of major plant proteins such as Ribulose-1,5-bisphosphate carboxylase/oxygenase (RuBisCo) from the purified product. After that, different purification strategies to intensively recover highly stable and purified VLPs were tested in this study. Therefore, combinations of CsCl density gradient analysis, ammonium sulfate precipitation, and different chromatographic purification techniques were employed to remove contaminated proteins and recover purified ones and eventually to standardize the best conditions for purification. Since VLPs were only partially purified when a single purification method was used, we used a combination of steps to remove contaminated proteins. The VLPs recovered after the purification steps were abundant and highly antigenic as evident by electron microscopic and ELISA analysis. Although the HPV-neutralization activity of plant VLP-induced antibodies was not assessed in the present study, their high binding titers to insect cell-produced VLPs, as shown in Fig 5B, suggest the efficacy of the plant-made immunogen. To prove this, a study is currently underway to compare the neutralization activities of sera from mice immunized with insect cell- and plant-produced VLPs.

Another problem in previous studies was the aggregation of proteins. Therefore, purification conditions that maintain the intact conformation of VLPs are important because recombinant VLPs are inherently unstable and tend to denature and aggregate in solution [12, 28]. To stabilize and protect HPV VLPs from aggregation and concentration loss, a series of commercially available non-ionic surfactants were previously evaluated [34]. As suggested, we used a minimal amount of polysorbate to increase the storage viability of VLPs and protect against aggregation. Notably, our experimental conditions proved that the presence of polysorbate 20 at a low concentration of 0.01% and salt conditions (ca. 0.1–0.2M) significantly enhanced the storage stability of VLPs during all of the purification steps.

AMS was used as an alternative method to purify L1 proteins. In agreement with a previous study [19], our results suggested that an AMS precipitation step is an effective method for removal of contaminants with the recovery of most of the soluble L1 proteins prior to the chromatography step. However, this method did not produce VLP formation of L1 monomers or capsomers. Similarly, low amounts of L1 proteins were obtained from the cation column because most of the L1 protein was washed away during the purification process. It has been suggested that the interaction between HPV16 VLP and heparin was dependent on a positive charge distribution. Although we used the cation column instead of a heparin column, a possible reason for losing the L1 protein could be the low positive charge distribution on its matrix as proposed by Knappe et al. [35]. More investigation is required to determine the feasibility of cation exchange chromatography to purify plant-produced L1 VLPs.

In conclusion, we demonstrated that a magnlCON plant virus vector with full-length L1 and a chloroplast transit peptide successfully produced HPV16 L1 VLPs in tobacco plants. Extraction of HPV16 L1 from leaf tissue was most efficient (>2.5% of TSP) with a low-salt phosphate buffer in the presence of HPV16 L1 VLPs, predominantly 35–55 nm in size, evident from electron microscopic studies. Although we used CsCl density gradient centrifugation as the preliminary step to remove plant proteins and collect VLPs, these data may be useful for the development of chloroplast-based vaccines. We anticipate that our new purification methods described herein will aid in recovering highly antigenic HPV16 VLPs from tobacco plants towards the goal of reducing time, cost, and labor while increasing the capacity for industrial production.

## Supporting Information

S1 Fig Detection profile of non-infiltrated tobacco leaves. Immunoblot represents the detection profile of HPV16 L1 protein where NC; negative control corresponds to non-infiltrated tobacco leaves, V3; PBS extracted 6 DPI tobacco leaves with V3 construct and PC; positive control, VLPs derived from insect cells. MoAbs (16E and 1H8) and PoAb (disCPV2) were used to detect sequential epitopes of HPV16 L1 protein.
(TIF)

## Abbreviations

HPV: human papillomavirus
VLP: virus-like particle
TP: chloroplast transit peptide
AMS: ammonium sulfate
SEC: size exclusion chromatography
IEC: ion-exchange chromatography
DPI: days post infiltration
TSP: total soluble protein
MoAb: monoclonal antibody
PoAb: polyclonal antibody
