# Supplementary figures and images for: Scalable Production of HPV16 L1 Protein and VLPs from Tobacco Leaves
Source: PLoS One. 2016 Aug 12;11(8):e0160995. doi: 10.1371/journal.pone.0160995 (PMC4982596; doi:10.1371/journal.pone.0160995)

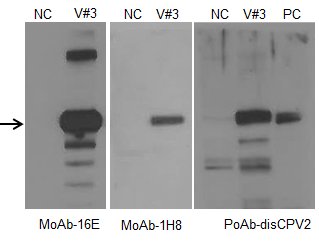

Supplement: S1 Fig — Immunoblot represents the detection profile of HPV16 L1 protein where NC; negative control corresponds to non-infiltrated tobacco leaves, V3; PBS extracted 6 DPI tobacco leaves with V3 construct and PC; positive control, VLPs derived from insect cells. MoAbs (16E and 1H8) and PoAb (disCPV2) were used to detect sequential epitopes of HPV16 L1 protein. (TIF) [file pone.0160995.s001.tif]
